# Evaluating the effect of high-intensity focused ultrasound therapy on liver tumors using multislice CT perfusion

**DOI:** 10.3892/ol.2012.1068

**Published:** 2012-12-10

**Authors:** XINSHAN CAO, XINGYUE JIANG

**Affiliations:** Radioactive Department, Affiliated Hospital of Binzhou Medical College, Binzhou, Shandong 256603, P.R. China

**Keywords:** high-intensity focused ultrasound, liver tumor, multislice CT perfusion, animal model

## Abstract

The aim of this study was to investigate the changes of the multislice computed tomography (MSCT) perfusion parameters and histopathology of the liver in rabbits with VX2 tumors before and after high-intensity focused ultrasound (HIFU) therapy. VX2 carcinoma cells were implanted into the livers of eight New Zealand white rabbits 3 weeks prior to the treatment. MSCT perfusion was performed one week before and one and six weeks after the treatment. These CT perfusion (CTP) data, including hepatic blood flow (HBF), hepatic blood volume (HBV), mean transit time (MTT) and permeability-surface area product (PS), were analyzed semi-quantitatively and qualitatively. Furthermore, the histopathological features of the liver tissues were also assessed semi-quantitatively before and after the treatment. Six weeks after HIFU therapy, MTT increased noticeably from 5.45±0.27 to 10.38±2.22 sec (P<0.05) and PS decreased significantly from 79.03±3.41 to 68.13±0.21 ml/100 g/min (P<0.05), while no significant differences in HBF and HBV were found. Furthermore, more CD3^+^ T cells were observed at the rim and center of the liver tumors six weeks after treatment. Therefore, HIFU therapy may be a simple and effective method for the treatment of liver tumors. CTP, as an effective method to obtain functional information about HBF, is able to quantify tumor vascularity and angiogenesis in liver tumors.

## Introduction

The formation of hepatocellular carcinoma (HCC) is usually sporadic, but the increased incidence of HCC is associated with familial disease syndromes such as familial adenomatous polyposis (FAP) and Beckwith-Wiedemann syndrome (BWS) (1). High-intensity focused ultrasound (HIFU) is, similar to laser treatment, a highly effective tool due to its minimimally invasive properties and accurate location targeting. Within a few seconds, sound waves access and thermally coagulate the target tissue to a certain depth below the skin, while sparing the surrounding tissue areas. HIFU therapy allows the recording of an accurate definition of the target area and the monitoring of therapy in real time. The aim of the HIFU therapy is the complete denaturation of the localized tumor tissue. It is believed that the cells are irreversibly damaged when heated to above 60°C after a few seconds (<8 sec) regardless of the tissue type. HIFU therapy, as an alternative treatment, has been used for VX2 liver tumors in the rabbit, in which tumors have directly invaded the local prostate tissue (2).

Computed tomography perfusion (CTP), as a tool to obtain functional information about blood flow, efficiently locates abnormal tissue perfusion which is difficult to detect accurately with conventional CT and MRI (3). CTP may be performed quickly and provide valuable data for diagnosis. The present study aimed to investigate the changes of the multislice CT (MSCT) perfusion parameters and histopathology of the liver in rabbits with VX2 tumors before and after HIFU therapy.

## Materials and methods

### Animal model

Eight New Zealand white rabbits weighing 4–5 kg were used in this study. As the blood supply of the VX2 tumor is similar to that of human HCC (4,5), VX2 carcinoma cells were used. VX2 cells were implanted into the hind limb of a donor rabbit and grew in the hind limb. When the size of the tumors reached 7–8 mm^3^ in volume, the tumors were harvested and implanted into the livers of eight rabbits 3 weeks prior to the HIFU treatment. The VX2 liver tumors grew in all eight rabbits. All animals received humane care in compliance with the Principles of Laboratory Animal Care formulated by the National Society of Medical Research and the Guide for the Care and Use of Laboratory Animals, published by the US National Institutes of Health. The protocol was approved by the Animal Care and Use Committee of Binzhou Medical University.

### HIFU therapy

The Haifu Model JC HIFU system (Chongqing HAIFU Company, Chongqing, China) was used as described previously (6). Briefly, the device is equipped with a 12-cm diameter, single element piezo-ceramic transducer fronted by acoustic lenses of varying focal lengths. An AU3 US imaging device (Esaote, Genoa, Italy) is mounted coaxially with the high-energy transducer allowing treatment to be guided in real time. Each rabbit underwent two HIFU treatment sessions under general anesthesia. Treatment consisted of a combination of single and multiple overlapping ultrasonic pulses directed to the target liver tumor. According to the protocols, a single tumor, or part of a single tumor, was selected for ablation.

### CTP imaging

CT scans were performed in the transverse plane using a 64-channel multidetector CT scanner (Sensation Cardiac 64). CTP consisted of a 60-sec series with 30 gantry rotations performed in cine mode during the intravenous administration of iodinated contrast material. Images were acquired and reconstructed at a temporal sampling rate of 1 image per 2 sec, resulting in a series of 20 images for each assessed section. Following unenhanced CT of the whole liver, eight adjacent 7.2-mm-thick sections were selected by starting at the level of the basal ganglia. A test bolus of 40 ml Ultravist 370 (Schering Health Care Ltd., Berlin, Germany) was administered into a vein, and saline chaser bolus of 20 ml was administered using a power injector at an injection rate of 5.0 ml/sec. At 4 sec after initiation of the contrast injection, a cine scan was initiated with the gantry angle parallel to and above the orbital roof to avoid radiation exposure to the lens. Dynamic CTP scanning was performed on the four-layered region including the circle of Willis for 40 sec with 7.2 mm thickness and 7.2 mm coverage area.

### Data analysis

Post-processing was performed using Siemens perfusion CT software. The software relied on the central volume principle to calculate perfusion parameters from the time-concentration curve. The highest peak was selected as the output vein and combined with time-density curves (TDCs) of contrast agent through the tissue to obtain the hepatic blood flow (HBF), hepatic blood volume (HBV), mean transit time (MTT) and permeability-surface area product (PS). The abnormal perfusion regions were observed to find the changes of distribution and color. The abnormal perfusion regions were viewed as regions of interest (ROI) if the surroundings demonstrated pathological changes, these pathological regions were also included in the ROI. When outlining ROI, it was important to avoid the great vessels. Four consecutive absolute CTP data were obtained by the mirror method.

### Histology/immunohistology

The histopathological features of liver tissues were assessed semi-quantitatively before and after the treatment. The liver tumor samples were collected from rabbits before and after the treatment. The samples were conserved in 10% buffered formalin, and 5-μm-thick sample sections were prepared for hematoxylin and eosin (H&E) staining to evaluate the basic histomorphology of the specimens. Immunohistochemistry was performed using a rat anti-CD3 antibody (pan-T-cell marker; Serotec, Oxford, UK), which shows a wide range of species cross-reactivity (7), using the a streptavidin-biotin detection system (Super Sensitive Immunodetection System; Biogenex, San Ramon, CA, USA) as previously described (8).

### Statistical analysis

Comparisons of CTP data between before and after surgery were performed using a Student’s t-test. The data are presented as mean ± SD. P<0.05 was considered to indicate statistical significance. Data were analyzed with the SPSS 18.0 statistical software package (SPSS Inc., Chicago, IL, USA).

## Results

**Figure 1.**
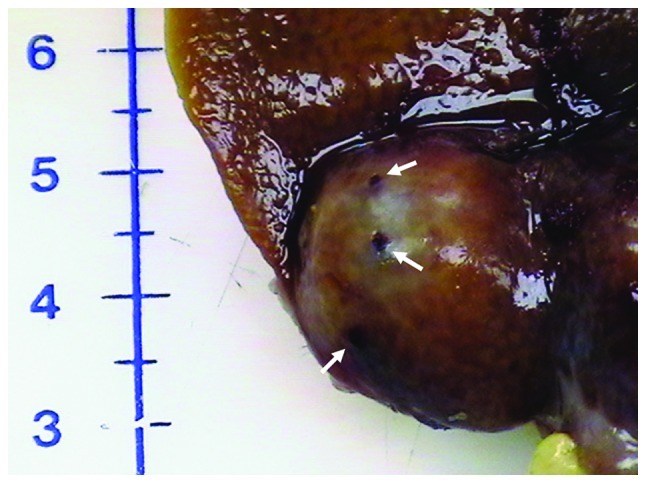
Image of rabbit VX2 liver tumor.

The implanted VX2 liver tumors grew successfully in eight rabbits. The size of the tumors ranged from 1.0 to 2.3 cm in diameter. All tumors were successfully catheterized and a region of hypervascular was visualized using digital subtraction angiography (DSA). As expected, hypervascular phenomenon was generally higher in the most viable, peripheral portion of the tumor. On ultrasound, the liver VX2 tumors appeared iso- to hypoechoic. Livers of the rabbits were biopsied successfully before and after HIFU treatment with a needle biopsy under ultrasound guidance. There were no any complications during the procedures and no signs of heavy bleeding or arteriovenous fistula were observed in the harvested tumors after HIFU treatment (Fig. 1).

**Figure 2.**
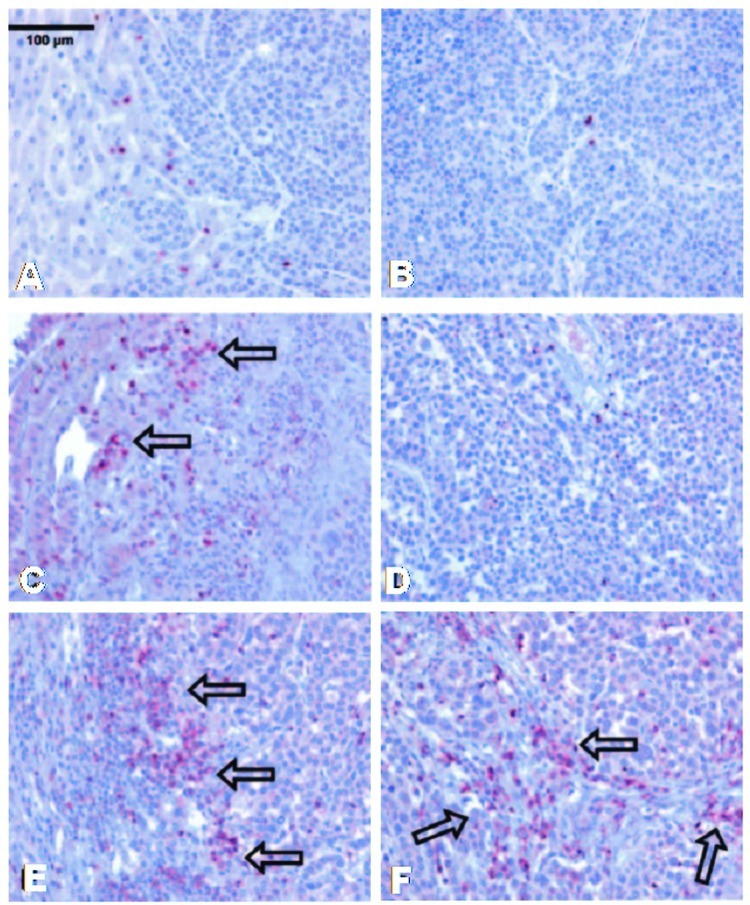
Immunohistochemical analysis for rabbits with VX2 liver tumors before and after HIFU therapy. Margin (A) and center (B) of a VX2 liver tumor before therapy. CD3^+^ lymphocytes (arrows) in the margin (C) and center (D) of a tumor one day after HIFU treatment. CD3^+^ lymphocytes (arrows) in the margin (E) and center (F) of a tumor three weeks after HIFU treatment. Magnification, ×200.

**Figure 3.**
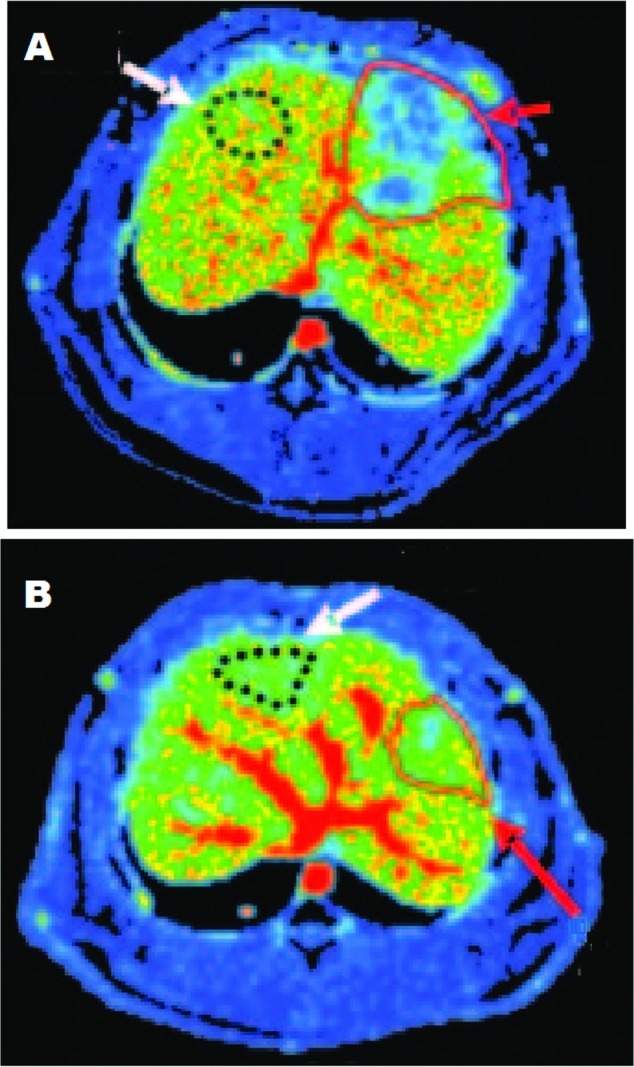
HBF maps obtained by CT perfusion (A) 1 week before and (B) 6 weeks after therapy (the normal tissue: white arrows; tumors: red arrows). HBF, hepatic blood flow; CT, computed tomography.

Results of immunohistology revealed that few CD3^+^ lymphocytes were present in the liver tumor before HIFU treatment (Fig. 2A and B). One day after HIFU treatment, more CD3^+^ lymphocytes were observed in the hemorrhagic margin around the tumor (Fig. 2C). Typical signs of cytoplasmic and nuclear changes were also observed in tumor cells following HIFU treatment. Here, infiltration of CD3^+^ lymphocytes was rarely found, however, there were still more than prior to HIFU treatment (Fig. 3D). Three weeks after HIFU treatment, more CD3^+^ T cells were observed not only in the margin between the normal tissue and the tumor (Fig. 2E), but also in the middle of the tumor (Fig. 2F).

HBF maps obtained by CT perfusion were compared at 1 week before and 6 weeks after therapy to show the change of these functional CT parameters during the growth of the tumor (Fig. 3).

Six weeks after HIFU therapy, MTT increased noticeably from 5.45±0.27 to 10.38±2.22 ml/100 g/min (P<0.05) and PS decreased significantly from 79.03±3.41 to 68.13±0.21 ml/100 g/min (P<0.05). HBF decreased from 265.53±5.26 to 256.97±8.07 ml/100 g/min, while HBV decreased from 21.91±1.38 to 20.85±1.27 ml/100 g. However, no significant differences in HBF and HBV were found (Table I).

## Discussion

Phenomenon has indicated that focused ultrasound may activate tumor-specific immune response. However, the physical properties of ultrasound limits the application of HIFU in the air-filled organs (lung, stomach, intestines, gall bladder, pancreas and parts of the esophagus) and those that are covered by bone (thoracic organs). The absorption of these interfaces may also lead to unwanted damage to adjacent organs (9,10). In cases of insufficient coupling, a similar mechanism leads to redness and burning of the outer skin (11,12). Another problem is the shifting of abdominal organs by respiratory motion. The organs may be shifted by up to 20 mm within a respiratory cycle and this significantly limits the efficiency and safety of the procedure. A solution to this problem can only be provided by reliable real-time imaging, which provides direct control of focus localization. Ideally, this would also be integrated into an automatic readjustment to compensate for the respiratory amplitude in the treatment system (13), although this technique has not yet been fully developed.

The theoretical possibility has been discussed that the spread of tumor cells could be inhibited through the mechanical effects of focused ultrasound (9). Studies of this issue, however, have shown that the metastatic rate is not increased by treatment with HIFU (14–16). In addition, several research groups have revealed an inhibitory effect on the growth of existing metastases, a reduction in the development of metastases or even regression of existing metastases (16–19). These phenomena may be associated with the activation of endogenous antitumor immunity. According to this theory, tumor cells rupture and fragments of the cells act as specific antigens. Wu *et al* showed a significant increase in CD4^+^ lymphocyte population after the HIFU treatment of osteosarcoma and renal cell carcinoma, and it may be associated with an activated systemic cellular immune response (15). The first indications of this phenomenon were reported by Wagai and Kaketa in 1970, who revealed that tumor-bearing rats had significantly higher rates of resistance against proliferating tumor cells following treatment with HIFU (17). Wu *et al* also showed that cancer cells exhibit malignant characteristics, including invasiveness, unregulated growth, metastasis and immortality, after HIFU treatment (18). This may prove to be a great advantage over conventional surgery, in which excessive growth of metastases following resection of the tumor itself is often observed. The reason for this may be the release of growth factors, an imbalance of pro- and antiangiogenic factors or the general immunosuppression following surgical intervention (20,21).

CTP data processing that can typically be achieved in 5 min is performed with postprocessing software using either rate-of-upslope estimation of HBF or deconvolution analysis. Only deconvolution analysis leads to quantitatively accurate results, including in areas with low perfusion (22). Generally, absolute values of CTP parameters can be calculated by CTP software, such as CT Perfusion 2 (23,24). However, Leenders *et al* found that CTP parameters varied in a large range due to individual differences and the experience of operational staff (25). Comparing HBF, HBV, MTT and PS values between abnormal regions and mirror-image control regions is an effective method of measuring the status of underperfusion present in a given case or location.

In summary, our findings suggest that HIFU therapy may be a simple and effective method for the treatment of liver tumors. CTP, as a smart method to obtain functional information about HBF, is able to quantify tumor vascularity and angiogenesis in liver tumors.

## Figures and Tables

**Table I.** Comparison of HBF, HBV, MTT and PS between 1 week before and 6 weeks after therapy.

CTP data	Preoperative 1 week	Postoperative 6 weeks	T-value	P-value
HBF, ml/100 g/min	265.53±5.26	256.97±8.07	0.845	0.442
HBV, ml/100 g	21.91±1.38	20.85±1.27	0.631	0.131
MTT, sec	5.45±0.27	10.38±2.22	−6.687	0.002^a^
PS, ml/100 g/min	79.03±3.41	68.13±0.21	5.704	0.011^a^

Data are presented as the mean ± SD;

a Values are statistically significant (P<0.05). CTP, computed tomography perfusion; HBF, hepatic blood flow; HBV, hepatic blood volume; MTT, mean transit time; PS, permeability-surface area product.
